# Cognitive remediation therapy plus behavioural weight loss compared to behavioural weight loss alone for obesity: study protocol for a randomised controlled trial

**DOI:** 10.1186/s13063-017-1778-x

**Published:** 2017-01-26

**Authors:** Evelyn Smith, Charlotte Whittingham

**Affiliations:** 1 Eating Disorders and Obesity Psychology Research Clinic, School of Social Sciences and Psychology, Western Sydney University, Locked Bag 1747, Penrith 2751, Sydney, NSW Australia; 2 Clinical and Health Psychology Research Initiative, School of Social Sciences and Psychology, Western Sydney University, Locked Bag 1747, Penrith 2751, Sydney, NSW Australia

**Keywords:** Cognitive remediation therapy, Executive functioning, Obesity, Binge-eating, Inflammation

## Abstract

**Background:**

Current research indicates that obese individuals have cognitive deficits in executive function, leading to difficulties with planning, impulse control and decision-making. High levels of inflammation have been proposed to contribute to executive function deficits in individuals with obesity.

**Methods/design:**

One hundred and seventy-six obese participants will be randomly assigned to one of two groups: (1) behavioural weight loss alone (BWL) group = 8 sessions of individual BWL sessions plus 12 group BWL sessions or (2) Cognitive Remediation Therapy for Obesity (CRT-O) plus BWL group (CRT-O + BWL) = 8 sessions of individual CRT-O plus 12 group BWL sessions. The study is double blind – participants will only be told that two weight-loss treatments are being compared and research assistants conducting outcome assessments will not know participants’ group allocation. Blood tests will be conducted to measure inflammatory markers. Measurement points will be at baseline, post treatment and 1-year follow-up. The primary outcomes will be differences between treatment groups in percentage weight loss, executive function, binge eating and an examination of whether changes in executive function predict changes in weight and binge eating. Secondary outcome measures will examine changes on inflammation, quality of life, and grazing behaviour and whether these predict changes in executive function and weight.

**Discussion:**

If CRT-O + BWL is more effective in assisting people to lose weight long term than BWL alone it should significantly improve treatment outcomes. This study expands upon our recent trial which showed that CRT-O enhanced executive function and weight loss in obese adults. The current study is strengthened by several factors: it is double-blind, it uses an active control, has a larger sample size, and measures inflammation to examine the mechanisms.

**Trial registration:**

The RCT is registered with the Australian New Zealand Registry of Clinical Trial, trial identifier: ACTRN12616000658415. Registered on 20 May 2016.

**Electronic supplementary material:**

The online version of this article (doi:10.1186/s13063-017-1778-x) contains supplementary material, which is available to authorized users.

## Background

According to the World Health Organisation more than 1.9 billion adults are overweight and of these 600 million are obese [1]. Between 1980 and 2014, the prevalence of obesity has more than doubled [1]. In Australia, the economic costs of obesity have been estimated at AU$21 billion a year [2]. Furthermore, the disability associated with obesity and its consequences has been calculated at over 36 million disability-adjusted life years, due mainly to ischemic heart disease and type 2 diabetes [3]. The comorbidities related to obesity, such as type 2 diabetes and coronary heart disease, however, are alleviated by modest weight loss [4] and long-term maintenance of the reduced weight [5]. Recommendations for obese individuals include losing 5–10% of body weight, and maintaining the loss, in order to reduce the risk of developing chronic medical conditions [6]. Whilst a number of current interventions assist individuals to reduce 5–10% of their body weight, most people regain the weight once professional contact ends [7]. Therefore, new treatment interventions which assist with weight-loss maintenance are urgently needed.

Recent research has examined the role of executive function in individuals with obesity. Executive function refers to a range of higher-order cognitive processes that facilitate initiation, planning, inhibition, cognitive flexibility, working memory, self-regulation, sequencing, and the achievement of complex, goal-oriented behaviour [8]. Obesity has been linked to deficits in executive function. A review of 24 studies found highly consistent results, with 22 studies showing a negative association between obesity and executive function [9]. This finding remained consistent even after controlling for age, sex, education, socioeconomic status, smoking, and cardiovascular and metabolic markers. Obese individuals with and without binge-eating disorder (BED) have been shown to have similar cognitive deficits [10]. In combination, these findings support a link between obesity and deficits in executive functioning, irrespective of common psychological or medical comorbidities.

The exact mechanism through which obesity and cognitive function are linked remains unknown. Smith and colleagues [9] have argued that a bidirectional relationship between executive function and obesity may be present whereby obesity impairs executive function via biological mechanisms, such as glucose abnormalities or inflammatory markers, and deficits in executive function exacerbate weight gain by causing difficulties with the regulation of food intake, and an inability to plan ahead or inhibit responses or a tendency to act impulsively. Research supports the notion that executive functioning is an important predictor of healthy eating, breakfast consumption and the inhibition of alcohol consumption [11–13] which are all predictors of obesity. Targeting these deficits in the obese may potentially improve a person’s response to therapy. This line of enquiry has led to the development of a new form of treatment for weight disorders, namely Cognitive Remediation Therapy for Obesity (CRT-O) [9, 14].

CRT-O is a manualised, face-to-face treatment that aims to improve underlying executive function deficits. It consists of mental exercises aimed at improving cognitive strategies, thinking skills and information processing through practice [14]. CRT for Anorexia Nervosa (CRT-AN) has been shown to be effective at improving executive function and eating-disorder-related quality of life (post treatment) and reducing psychopathology (at 6-month follow-up) [15]. Unlike CRT-AN, CRT-O is intended to assist people to maintain weight loss and prevent weight gain by associating cognitive style to food intake and exercise behaviours.

Our recent randomised controlled trial (RCT) on CRT-O for obesity [16] provided evidence for the efficacy of CRT-O. Eighty obese individuals received three 90-min sessions of behavioural weight loss (BWL: a standard weight-loss treatment) and were then randomised to either eight sessions of individual face-to-face CRT-O or a no-treatment control condition. Results showed that those in the CRT-O group experienced a significant improvement in executive function and a significant decrease in weight compared to controls, both with large effect sizes, and that changes in executive function predicted changes in weight. Participants also had a significant reduction in binge eating in the CRT-O condition, compared to controls. This highlights that CRT-O can improve executive function and help individuals to manage their weight. The data from this trial suggests that CRT-O could enhance weight-loss outcomes, without targeting eating or exercise behaviour directly.

Our present RCT will endeavour to determine the effectiveness of eight individual sessions of CRT-O plus 12 group sessions of BWL compared to eight individual and 12 group sessions of BWL alone (standard care) in obese patients, in terms of reducing weight, binge eating, grazing, and improving executive function and quality of life. In addition, as high levels of inflammation have been proposed to contribute to executive function deficits in obese individuals, inflammatory markers will be assessed to test this hypothesis [17]. We hypothesise that those who receive CRT-O plus BWL will show greater weight loss, improved executive functioning and quality of life, and reduced binge eating/grazing compared to individuals in the BWL alone condition at post-treatment and 1-year follow-ups.

## Methods/design

### Participants

Participants will be 176 individuals, aged 18–55 years, with a Body Mass Index (BMI) ≥30.0 kg/m^2^ who can provide informed consent, have sufficient English skills, and are able to attend treatment on campus. Participants are excluded if they have been diagnosed with somatic conditions (e.g. neurological disorders, stroke, head injury); serious psychiatric/psychosomatic conditions (e.g. psychotic disorder, suicidality, severe substance use disorder, developmental or intellectual disability); impediment in hearing, vision, or language with an effect on testing; previous or planned bariatric surgery; use of medication that impacts weight or executive functioning (e.g. antipsychotics, sedatives). These inclusion and exclusion criteria will allow for recruitment of a representative study sample, heterogeneous with regard to body weight, degree of obesity, and prevalence of typical comorbidities.

### Procedure

Participants will be recruited from clinic settings and the community, with close to equal numbers of male and female subjects (although more female subjects are anticipated based on previous trials). Advertising will also be placed on social media sites, community notice boards and newspapers. Participants will be asked to provide their contact details if they are interested in taking part in the study. The research psychologist will telephone or email the participant to make contact and to inform them about what is involved. If the participant is interested in taking part, the psychologist will conduct a screening interview to assess whether the potential participant is suitable and will schedule an assessment time.

This is a double-blind study; participants are blind to the design of the study, i.e. they are not provided with any information regarding the CRT-O intervention prior to randomisation and research assistants are blind to the participants’ group allocation at post-treatment and follow-up assessments. Participants are informed that two different weight-loss treatments are being compared. Participants will be required to have a GP who is responsible for their medical management during the trial. Participants will be randomly assigned to one of two groups using computer-generated random numbers, provided in a document developed by a researcher not in charge of recruitment or assessment:

1. BWL alone group = 8 sessions of individual BWL plus 12 sessions of group BWL or
2. CRT-O + BWL group = 8 sessions of individual CRT-O plus 12 sessions of group BWL

The RCT has been registered with the Australian New Zealand Registry of Clinical Trial (trial id: ACTRN12616000658415). The trial followed the SPIRIT guidelines (see additional file 1).

### Behavioural weight-loss intervention (BWL)

Those in the BWL group will attend eight individual sessions with a psychologist, where they will receive extensive education about weight-loss techniques and receive support towards achieving their weight-loss goals including managing emotions, cravings and establishing hunger cues. Following this, they will attend 12 weekly (2-h) BWL group sessions which will teach them how to change their diet and exercise habits to enable weight loss. Treatment requires participants to record their diet and exercise behaviour in a diary.

During these sessions the psychologist will reinforce the BWL strategies and help the participant to problem-solve any difficulties that they are having with their implementation. The group programme is a modified version of the Look Ahead trial [18, 19]. The Look Ahead trial is an intensive lifestyle intervention programme that targets diet and exercise through behavioural modification techniques and has been extensively researched [18, 19]. The programme has been modified to include Australian dietary and physical activity guidelines. Interestingly, BWL programmes have been found to be more successful at enhancing weight loss than cognitive behavioural therapy [20].

During BWL, behavioural techniques are used to help participants modify eating and exercise habits. A dietitian will conduct an early BWL group session regarding dietary advice. Participants are taught to manipulate their environment at home and at work by limiting cues associated with eating and increasing cues associated with exercise. For example, participants are encouraged to keep high-fat, high-calorie foods out of their house and work area. Problem-solving techniques are taught so that participants can effectively deal with difficult situations that threaten their weight-control efforts. Thoughts and emotions related to overeating and inactivity are also addressed during treatment sessions to understand and decrease the influence of these factors on weight control. Stress management, craving exposure and mindful eating techniques will be taught and attention is given to motivation enhancement and relapse prevention to help individuals to maintain their weight loss. Homework will also be provided.

### Cognitive Remediation Therapy for Obesity (CRT-O)

CRT-O is a manualised intervention that consists of mental exercises aimed at improving executive function via practice [14]. CRT promotes reflection on thinking styles, develops metacognition and helps to explore and apply new thinking strategies in everyday life. The primary function of CRT is to improve the thinking process rather than the content [21–23]. A manual was developed for the pilot CRT-O trial. The current trial will also utilise this manual. The CRT-O treatment will be delivered face-to-face by two clinical psychologists. The main components and principles of CRT-O are based on the original CRT-AN treatment. However, some important modifications have been made to adapt the manual for obesity and these are described in Smith et al. [14]. Homework will also be provided.

### Assessment

Participants will complete a comprehensive assessment at baseline, at post-treatment and 1-year follow-ups consisting of:

1. *Demographics*: age, sex, ethnicity/race, socioeconomic status (by postcode, income, education), and language use
2. *Anthropometric measures*: we will measure: (1) height and weight with calibrated scales (to derive BMI in kg/m^2^), (2) hip and waist circumferences to calculate the waist to hip ratio, and (3) we will use a portable bioelectrical impedance scale (from Tanita) to calculate an estimate of total body water, which can then be used to estimate fat-free body mass and, by difference with body weight, body fat. (One study showed that Tanita and DEXA measures correlated at 0.96 [24]). Weight will be measured at every session
3. *Exercise*: participants will wear an accelerometer for 1 week (at three time points) to assess physical activity levels. Accelerometers are effective tools that continuously record physical activity over specified time intervals. The data output also allows for an estimate of physical activity intensity and energy expenditure (which are not possible with alternative methods, such as pedometers). To not over-burden participants during assessment (week 0), the accelerometer will be provided in week 1 of the intervention
4. *Neuropsychological assessment*: a targeted assessment of cognitive function will be completed at the time of their preference. It will include:
*Executive function:* the *Wisconsin Card Sorting Test* (WCST)-64: the computerised version of the WCST [25, 26] will be used. In this test, subjects are instructed to categorise a series of cards according to one of three stimulus features (colour, shape or number), without this principle being revealed to the subject. After each association, the only feedback given is whether a match is correct or incorrect, the idea being that subjects should infer how to categorise based on the feedback they receive. This test measures categorisation, inference, testing of hypotheses, cognitive flexibility, cognitive inhibition and response to feedback [27]. The number of total errors and perseverative errors and failure to maintain a set are the dependent measures. The WCST is regarded as a valid measure of abstract reasoning ability to maintain and appropriate planning and problem-solving strategies across changing stimulus conditions to obtain a future goal; it is the most widely used test of executive function [28]
*Trail Making Test* (TMT) [29]: this will be used to assess psychomotor speed, visual integration, cognitive flexibility and inhibitory control. This test measures the subject’s ability to connect written numbers in an ascending order (Trail A), and, afterwards, to connect numbers and letters, alternating numbers in ascending order and letters in alphabetical order (Trail B), for example, 1-A-2-B. The score is the response time taken to complete the respective trails [28]
*Behaviour Rating Inventory of Executive Function* (BRIEF-A): participants will complete the BRIEF-A Self-report Form. This instrument is a 75-item standardised measure that records views of an adult’s executive functions or self-regulation in a person’s everyday environment. It has been shown to be a reliable and valid measure of executive functioning in individuals across a broad age range [30]
*Memory: Wechsler Memory Scale* (WMS), *Logical Memory Subtest*: the Logical Memory Test is the most widely used measure of memory [31], and it targets auditory-linguistic memory. The task demands verbal recall of spoken story passages
*Language: Boston Naming Test* (BNT): the BNT is a 60-item confrontation naming test which is considered to be a reliable measure of word retrieval performance [32]
5. *Nutrition*: a comprehensive assessment, using a 1-week food diary, will be conducted by a dietitian and will be analysed using FoodWorks software. The Foodworks Professional package includes food composition tables and contains nutrient values for more than 4200 foods, beverages and supplements. FoodWorks is suitable for dietary analysis and meal planning
6. *Inflammatory markers*: tumor necrosis factor-α (TNFα), interleukin-6 (IL-6), inflammasome-activated IL-1β and C-reactive protein will be measured
7. *Clinical assessment*: a brief clinical assessment will verify comorbidities such as an eating disorder, depression or anxiety. The participant will also be asked questions related to their previous weight-loss attempts and beliefs about weight loss. The following measures will also be completed:
  - *Depression, anxiety and stress*: will be measured by the 21-item *Depression, Anxiety and Stress Scale* (DASS) [33], a well-validated Australian measure. It has been validated in clinical and nonclinical populations and has good reliability [33, 36]. Research has indicated that individuals who are obese are at an increased risk of developing depression and depression has been found to be predictive of obesity [34]. Also, the association between depression and obesity has been found to have a dose-response gradient where the association between depression and obesity has been found to be stronger than that between depression and being overweight [35]. There is a significant evidence base that indicates that depressive disorders are associated with deficits in cognition, including difficulties with verbal learning and memory [37], attentional functions, executive control, lowered cognitive flexibility [38] and interpretation biases [39, 40]
  - *Eating disorder symptoms*: eating disorder symptoms are to be measured by the *Eating Disorder Examination Questionnaire* (EDE-Q) [41]. The EDE-Q has 36 items and measures concerns about shape, weight and eating; restraint and self-reported binge eating. Subscale scores for shape, weight and eating concerns and restraint range between 0 and 6. A higher score indicates more severe eating psychopathology. This has been validated in community samples in Australia and it has been shown to hold acceptable internal consistency and test-retest reliability [42]. The BED and purging subsections of the EDE will also be administered
  - *Grazing Questionnaire*: grazing refers to ‘uncontrolled, repetitive eating of small amounts of food’. Grazing has been implicated as an eating behaviour which is associated with obesity. This measure is a 9-item self-report questionnaire which asks specific questions regarding eating behaviour [43]
  - *Quality of Life*: this will be assessed using the *Short Form-12 Health Status Questionnaire* (SF-12). This is a well-known and validated measure of functional limitations associated with impairment in physical and mental health

### Treatment fidelity

The clinical psychologists delivering the treatment will be trained and regularly supervised. Therapist adherence will be monitored through audio-recordings and monthly supervision sessions. Adverse events assessed by the psychologists will be reported to the Ethics Committee immediately. A psychologist can refer a participant to another psychologist for treatment of mental illness at any time. In addition, randomly selected taped sessions of CRT and BWL will be reviewed by two of the project’s principal researchers who will apply a previously developed Treatment Adherence Measure to the taped sessions. The Work Alliance Inventory (WAI) will be administered during weeks 4 and 8 of individual treatment to assess therapeutic alliance. In weeks 4, 8 and 12 of group treatment, the Group Climate Scale (GCS) will be provided to assess group factors. Session attendance will also be recorded.

### Data collection and analysis

Data is collected at baseline, post treatment and at 1-year post-treatment follow-up. See Table 1 for assessment tests and times of data collection. The Statistical Package for Social Sciences (SPSS) statistics will be used to analyse data. Data entry will be done and checked by two research assistants and stored in the university’s server. The principal researchers will conduct the analyses for the main results with assistance of trained statisticians. The Data Monitoring Committee is comprised of two researchers, one with expertise in cognitive remediation therapy and the other is the primary researcher (ES).

**Table 1:** Assessment tests and times of data collection for both groups

Assessments	Screening	Initial assessment (week 0)	Week 1 of treatment	Weeks 2–20 of treatment	Post treatment (week 20)	Follow-up (week 52)
Inclusion/exclusion criteria	X					
Demographic data		X			X	X
Anthropometric measures		X		X (at the end of one-to-one intervention)	X	X
Clinical assessment		X			X	X
Neuropsychological assessment		X			X	X
Nutrition data		X			X	X
Inflammatory markers		X			X	X
Accelerometers			X		X	X

#### Power calculations

We calculated the sample size with a method that takes into account the intracluster correlation coefficient (*r* = .02) for comparison between groups using a two-tailed linear mixed model, predicting a small effect size (Cohen’s *d* of 0.2) on weight loss and using a power of 80%, and determined that a sample size of 70 individuals per group is needed. Assuming an attrition rate of 25% per group, a sample of 88 per group is necessary, totalling 176 participants.

### Main analyses

Analyses for the main results will be conducted by the researchers with assistance from a statistician. Data will be inspected for normality and nonparametric statistics applied where appropriate. Percentage weight loss will be computed. Scores on cognitive measures will be transformed into z-scores. Linear mixed models will be used to compare differences between treatment groups in percentage weight loss, executive function, binge eating and to examine whether change in executive function predicted changes in weight and binge eating.

### Secondary analyses

To examine the baseline and prospective relationships between biological data and cognition/weight, linear or logistic regression analyses will be performed whilst controlling for potential confounders. The Bonferroni correction or FDR (False Discovery Rate) method will be used for multiple comparisons and post-hoc testing will be performed where appropriate. Secondary analyses for the other secondary measures will use either linear mixed models or repeated measure analysis of variance (ANOVA). We will report the main analyses in a separate publication to secondary analyses.

## Discussion

If CRT-O (plus BWL) is successful at assisting people to lose weight and to maintain the weight loss it would allow for the development of an intervention that could significantly improve treatment outcomes. Given the poor long-term outcomes for previously investigated treatments (excepting bariatric surgery reserved for the most severe cases) and the increasing public health burden of obesity, novel and integrative treatment approaches are urgently needed.

Our RCT is designed to compare two groups, a CRT-O (plus BWL) group to a BWL alone group. We will investigate whether CRT-O (plus BWL) is effective in improving a person’s ability to lose weight, to maintain their weight loss and to improve their executive functioning, compared to BWL alone. The strengths of our design include its large sample size, the use of validated and standardised assessments, and its double-blind study design. We will also be the first to measure inflammatory markers to discern whether there is an association between inflammation, weight and executive function in obese individuals.

### Trial status

Trial has started recruiting participants in September 2016.

## Additional file

Additional file 1: SPIRIT 2013 Checklist: recommended items to address in a clinical trial protocol and related documents*. (DOC 122 kb)

## Abbreviations

BED: Binge-eating disorder
BMI: Body Mass Index
BNT: Boston Naming Test
Brief-A: Behaviour Rating Inventory of Executive Function
BWL: Behavioural weight-loss intervention
CRT: Cognitive Remediation Therapy
CRT-AN: Cognitive Remediation Therapy for Anorexia Nervosa
CRT-O: Cognitive Remediation Therapy for Obesity
DASS: Depression, Anxiety, Stress Scale
EDE-Q: Eating Disorder Examination Questionnaire
GCS: Group Climate Scale
GQ: Grazing Questionnaire
SF-12: Short Form-12 Health Status Questionnaire
TMT: Trail Making Test
WAI: Work Alliance Inventory
WCST: Wisconsin Card Sorting Test
WMS: Wechsler Memory Scale
